# Damping ratio analysis of tooth stability under various simulated degrees of vertical alveolar bone loss and different root types

**DOI:** 10.1186/s12938-017-0388-x

**Published:** 2017-08-03

**Authors:** Kuo-Ning Ho, Sheng-Yang Lee, Haw-Ming Huang

**Affiliations:** 1 School of Dentistry, Taipei Medical University, 250, Wu-Hsing Street, Taipei, Taiwan; 2 Graduate Institute of Biomedical Optomechatronics, Taipei Medical University, Taipei, Taiwan

**Keywords:** Resonance frequency, Damping ratio, Tooth, Stability

## Abstract

**Background:**

The purpose of this study was to evaluate the feasibility of using damping ratio (DR) analysis combined with resonance frequency (RF) and periotest (PTV) analyses to provide additional information about natural tooth stability under various simulated degrees of alveolar vertical bone loss and various root types.

**Methods:**

Three experimental tooth models, including upper central incisor, upper first premolar, and upper first molar were fabricated using Ti6Al4V alloy. In the tooth models, the periodontal ligament and alveolar bone were simulated using a soft lining material and gypsum, respectively. Various degrees of vertical bone loss were simulated by decreasing the surrounding bone level apically from the cementoenamel junction in 2-mm steps incrementally downward for 10 mm. A commercially available RF analyzer was used to measure the RF and DR of impulse-forced vibrations on the tooth models.

**Results:**

The results showed that DRs increased as alveolar vertical bone height decreased and had high coefficients of determination in the linear regression analysis. The damping ratio of the central incisor model without a simulated periodontal ligament were 11.95 ± 1.92 and 27.50 ± 0.67% respectively when their bone levels were set at 2 and 10 mm apically from the cementoenamel junction. These values significantly changed to 28.85 ± 2.54% (*p* = 0.000) and 51.25 ± 4.78% (*p* = 0.003) when the tooth model was covered with simulated periodontal ligament. Moreover, teeth with different root types showed different DR and RF patterns. Teeth with multiple roots had lower DRs than teeth with single roots.

**Conclusion:**

Damping ratio analysis combined with PTV and RF analysis provides more useful information on the assessment of changes in vertical alveolar bone loss than PTV or RF analysis alone.

## Background

Good periodontal support with healthy periodontal conditions is important to provide protection against masticatory stress. Measurement of periodontal pocketing, X-ray examination, and in some cases, a histopathological examination is indispensable for the diagnosis of periodontal disease [1]. However, the estimation of pocket depth or attachment level by the probing technique is questionable due to its lack of accuracy [2, 3]. Radiographic analysis is another useful method for evaluating the quantity and quality of periodontal conditions, but a limitation of this method for diagnosing periodontal conditions is its low resolution. It is difficult to detect the bone status at the periodontal interface when demineralization is less than 30% [4, 5]. Since there is a strong correlation between the degree of tooth mobility and the loss of alveolar bone [6], measurement of tooth mobility can be used as a diagnostic parameter in evaluating periodontal tissue conditions.

The periotest (Siemens AG, Bensheim, Germany) was introduced as the first diagnostic device that measures tooth mobility objectively. The periotest can be used to detect not only tooth movement but also the early stages of ankyloses caused by dental trauma [7]. The device uses contact times between an acceleration rod and the target tooth surface as a parameter to represent the tooth mobility [8–10]. However, several investigators report that the periotest results do not always correspond precisely to biomechanical parameters because the periotest values (PTVs) are strongly related to the excitation direction, application force, and position [1, 11–13]. Recently, Goellner et al. detected teeth mobility of healthy individuals by both the periotest and a quantitative metric tooth mobility measuring method. They found no correlation between the metric displacement of the tooth and PTVs [14]. They suggested that besides the tooth displacement, the properties of the periodontal ligament also influence the PTV.

To overcome this problem, several researchers have tried to develop new techniques to analyze tooth mobility and periodontal tissue condition using different mechanical parameters. The resonance frequency is a vibrational parameter. Mathematically, the resonance frequency is a function of the stiffness and mass of a structure and is related to the boundary conditions of a vibrating object [15, 16]. In implant dentistry, the resonance frequency is also a useful diagnostic tool in the assessment of dental implant stability [17–20]. Lee et al. calculated the resonance frequencies of healthy upper central incisors using the finite element method and indicated that the resonance frequency was lower when the surrounding bone was decreased horizontally [21]. Based on this finding, Huang and colleagues measured the resonance frequencies of the anterior teeth in vivo [15, 16]. They found that the mean resonance frequencies of anterior teeth significantly decreased when periodontium attachment loss was greater than 4 mm. These findings suggest that resonance frequencies could be a useful parameter for assessing periodontal conditions clinically, such as the root length in alveolar bone [19] and molar furcation involvement [22]. However, until now, no resonance frequency device for detecting periodontal clinical conditions was commercially available. One possible reason for this lack of availability could be the low sensitivity of resonance frequency measurements for detecting periodontitis. In an in vivo study, Wang et al. showed that the difference in resonance frequencies between healthy teeth and teeth with periodontitis was only 6% [22].

The damping factor is another important parameter of vibration analysis. Damping is an effect that reduces the amplitude of vibrations in an oscillatory system. For nondestructive tests, damping is considered an important parameter for determining the viscoelastic behavior of composite materials by measuring energy loss. This loss of energy is known as the damping capacity [23]. In engineering, the damping effect can be expressed as the damping ratio, a dimensionless measure describing how oscillations of a structure decay after a disturbance. It is defined as the fraction of strain energy lost in one full cycle of deformation. Recent studies have demonstrated that the damping can be used as a noninvasive tool for monitoring changes in bone properties [24, 25], discriminating between varying degrees of osteoporosis and bone fracture [26], and diagnosing osseointegration during the dental implant healing process [27–29].

The periodontal ligament is a soft, richly vascular and cellular connective tissue that surrounds the roots of the teeth and joins the root cementum with the alveolar bone [30]. This structure distributes and absorbs forces produced during mastication and other types of tooth contact into the alveolar process via the alveolar bone proper. Thus, the periodontal ligament tissue acts as a damping mechanism that assists in the absorption of occlusal forces around the alveolar region of a loaded tooth [31]. Nonetheless, a device for determining the periodontal condition that directly measures the damping properties of a tooth remains unavailable.

Since successful treatment is based on a thorough examination, evaluation of tooth stability is an important diagnostic process that contributes greatly to treatment planning and clinical management. The aim of this study was to test whether the damping ratio provides useful information on tooth stability as the boundary conditions are decreased in teeth with various root types.

## Methods

### In vitro model setup

**Fig. 1:**
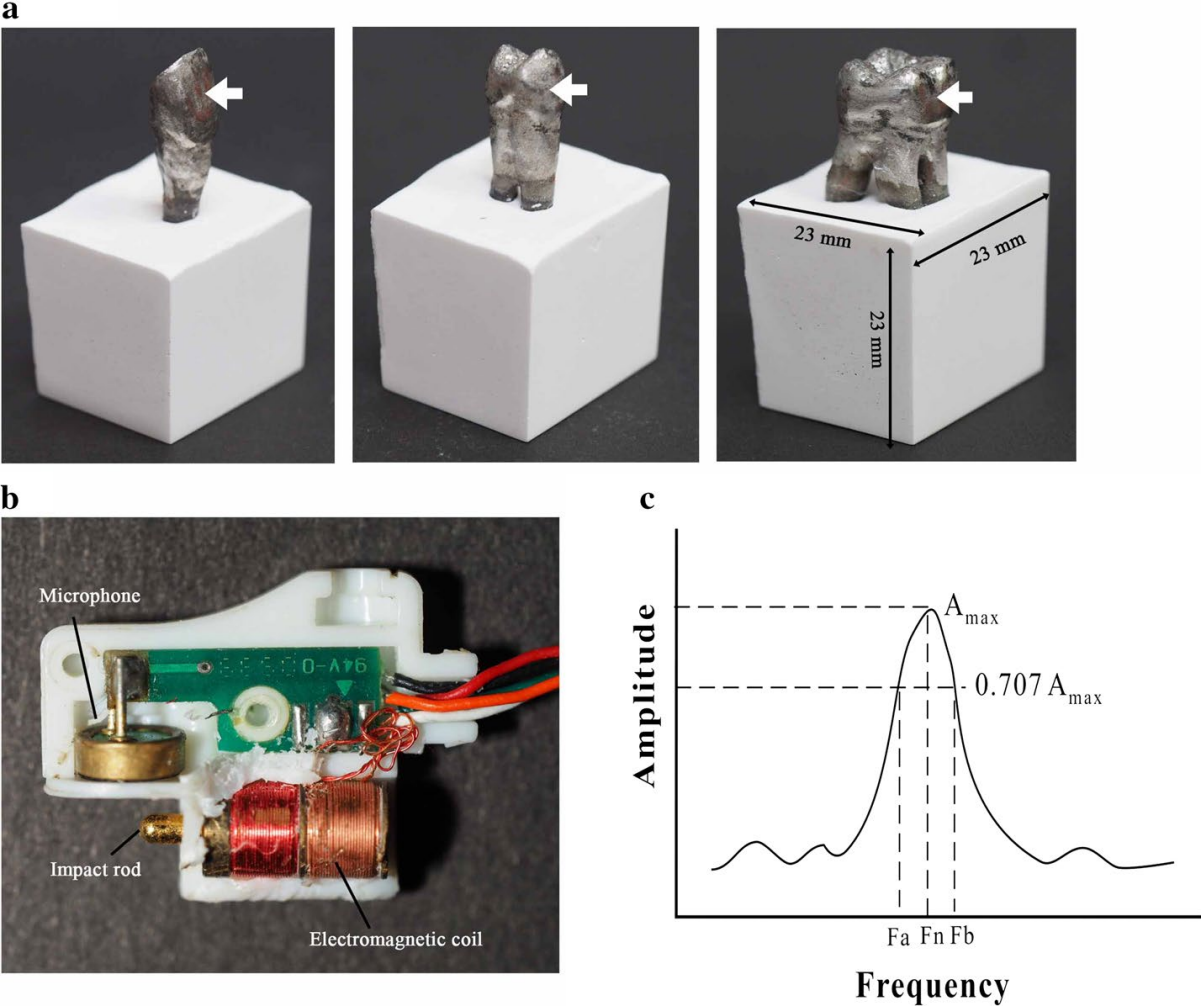
**a** The fabricated tooth models. The model setup with simulated tooth, periodontal ligament and alveolar bone. *White arrows* indicated the impact positions. **b** The detector probe consisted of a piezoelectric microphone and an impact rod, driven by electromagnetic coil. **c** The typical frequency response spectrum of the vibration experiment. Damping ratio was calculated using the half-power method. Fn, resonance frequency. Fa and Fb are the frequencies at 0.707 times the maximum amplitude of the resonance frequency

To evaluate the influence of different root types on the detected damping ratios, three types of teeth models, including upper central incisor, upper first premolar, upper first molar, with simulated periodontal ligaments, were fabricated (Fig. 1a). The model teeth were made of Ti6Al4V (115 GPa, 3D Printer Products, Chinmintai, Taipei, Taiwan). The shapes of the model teeth were adopted from standard teeth (Nissin Dental Products Inc., Kyoto, Japan). According to previous studies, a tissue conditioner for soft lining material (0.38 MPa, Shofu Tissue Conditioner II, Shofu Inc., Kyoto, Japan), and gypsum (1.76 GPa, Heraeus Kulzer GmbH, Germany) were used to simulate the periodontal ligament and alveolar bone, respectively [1, 32]. The thickness of the periodontal ligament of the simulated tooth models was 0.25 mm [33]. It was prepared according to previous work [34]. Briefly, root surfaces were dipped into melted wax (GEO Dip, Renfert GmbH, Germany) up to 2–10 mm below the CEJ. The 0.25 mm thick wax layer was confirmed with digital calipers. The roots of the teeth were embedded in gypsums in a plastic container (Fig. 1a). After gypsum set, the teeth were moved out from the gypsum and the wax was removed. Then tissue conditioner was used to fill the spaces between the simulated teeth and gypsums.

### Experimental procedures

In this study, a noninvasive impulse-forced vibrational method was used to determine the damping ratios of the tested teeth. As shown in Fig. 1b, the probe consisted of an impact rod and a piezoelectric microphone, as described previously [35]. When a current passed through an electromagnetic coil, the impact rod was driven forward by an electromagnetic force. In this study, central position on the tooth buccal surface was selected as impact point (Fig. 1a). The signal was detected by the microphone and sent to the spectrum analyzer (Implomate, Biotech One, Taipei, Taiwan). The frequency response was calculated using Fast Fourier Transform software. The specific resonance frequency (Fn) of the simulated teeth was determined from the highest point with a peak value of the vibration amplitude. The damping ratio was calculated using the half-power method (Fig. 1c) from the following formula [25, 33, 36]:

$${\text{DR}} = \frac{{({\text{Fb}} - {\text{Fa}}) }}{{2{\text{Fn}}}}$$

where Fn is the resonance frequency, (Hz) and Fa and Fb are the frequencies obtained from the points at 0.707 times the maximum amplitude of the resonance frequency. The formula used to calculate the damping ratio was justified in several previous in vitro studies [25, 33, 37].

To evaluate the influence of the simulated periodontal ligament on the test parameters, the damping ratio, resonance frequency were measured with a novel analyzer (Implomate, Biotech One, Taipei, Taiwan) which was described in previous research [29], and periotest (Siemens) were measured simultaneously [28] in conditions with various alveolar vertical bone loss in a tooth model with a single root (central incisor model) with and without a simulated periodontal ligament. In addition, the three parameters were measured in the three simulated teeth with various degrees of vertical bone loss simulated by decreasing the surrounding bone level apically from the cementoenamel junction in 2-mm steps incrementally downward for 10 mm [22, 38].

### Statistical analysis

Each measurement was obtained from three samples to verify its accuracy. The data are obtained from at least three separate measurements and are presented as means and standard deviations. A linear regression analysis was done to validate the detection technique and examine the relationships of the periotest value, RF, and the damping ratio in all tooth models with various simulated alveolar bone heights. Kruskal–Wallis and Dunn post hoc tests (SPSS, IBM) were used to assess the association between damping ratio and the conditions at the tooth-bone interface for various degrees of vertical bone height. p < 0.05 was considered to indicate statistical significance.

## Results

**Fig. 2:**
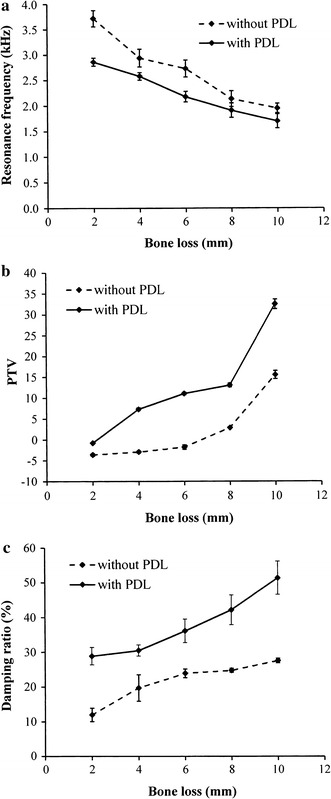
The relationship between the attachment level of the tested upper central incisor model and the **a** resonance frequency, **b** periotest value (PTV), and **c** damping ratio. PDL, periodontal ligament

**Fig. 3:**
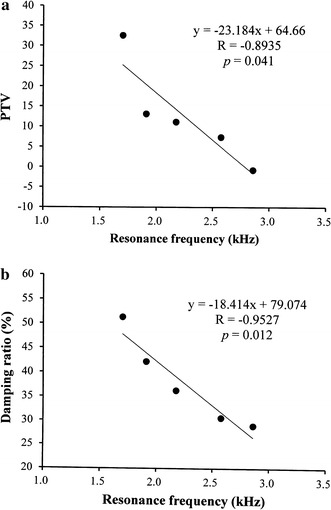
The relationship between the resonance frequency of the tested tooth model and the **a** periotest value and **b** damping ratio. A high correlation was found between the readings of resonance frequency and damping ratio

Resonance frequency, PTV, and damping ratio of the upper central incisors with and without periodontal ligaments were determined to evaluate the influence of the simulated periodontal ligament on the test parameters. For the tooth model without a simulated periodontal ligament, the RF, PTV, and damping ratio of the model with a bone level at 2 mm apically from the cementoenamel junction were 3716.67 ± 160.73 Hz (Fig. 2a), −3.62 ± 0.30 (Fig. 2b) and 11.95 ± 1.92% (Fig. 2c), respectively. When the amount of vertical alveolar bone loss increased from 2 to 10 mm, the RF decreased to 1950.00 ± 91.29 Hz (Fig. 2a) while the PTV and damping ratio increased to 15.57 ± 0.99 (Fig. 2b) and 27.50 ± 0.67% (Fig. 2c), respectively. The presence of periodontal ligament tended to decrease the resonance frequency (*p* = 0.026 < 0.05) and increase the PTV (*p* = 1.46 × 10^−5^ < 0.05) and increase damping ratio (*p* = 2.14 × 10^−10^ < 0.05) of the tooth models. When the upper central incisor model was covered with simulated periodontal ligament and embedded in the same simulated surrounding tissues, the detected RF, PTV, and damping ratio at 2 mm were 2858.33 ± 78.62 Hz (Fig. 2a), −0.78 ± 0.13 (Fig. 2b), and 28.85 ± 2.54% (Fig. 2c), respectively. These values changed to 1700.00 ± 136.93 Hz (Fig. 2a), 32.50 ± 1.17 (Fig. 2b), and 51.25 ± 4.78% (Fig. 2c) when the simulated bone levels of the tooth models were set at 10 mm apically from the cementoenamel junction. The PTV and damping ratio data were plotted against the analogous resonance frequency data. As shown in Fig. 3a, poor correlation was found between PTV and resonance frequency (R^2^ = 0.7984). However, a higher correlation coefficient was obtained between damping ratio and resonance frequency (R^2^ = 0.9077) (Fig. 3b).

**Fig. 4:**
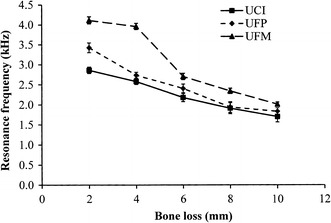
Changes in the resonance frequencies of the models with various root types and degrees of bone loss. *UCI* upper central incisor, *UFP* upper first premolar, *UFM* upper first molar

**Fig. 5:**
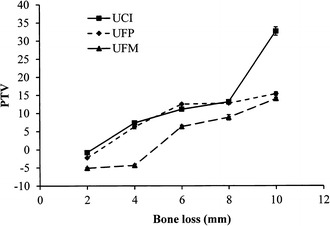
Changes in the periotest values (PTVs) of the models with various root types and degrees of bone loss. *UCI* upper central incisor, *UFP* upper first premolar, *UFM* upper first molar

**Fig. 6:**
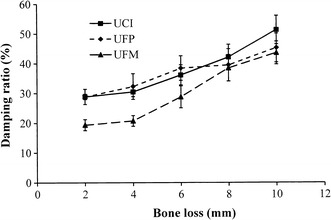
Changes in the damping ratios of the models with various root types and degrees of bone loss. *UCI* upper central incisor, *UFP* upper first premolar, *UFM* upper first molar

Figure 4 shows that regardless of the bone level, the upper first molar model had the highest resonance frequencies compared to the other two tooth models in all simulated bone loss situations. Among the three models, the upper central incisor exhibited the lowest resonance frequencies. For upper central incisor, upper first premolar, and upper first molar models, the fold changes in RF from 2 to 10 mm were 1.68, 1.86, and 1.87, respectively. For the periotests, the opposite trend was observed. The upper first molar showed the lowest PTV compared to the upper central incisor and upper first premolar models. A significant difference in PTV between the upper central incisor and upper first molar was observed only when the bone level was set at 10 mm apically from the cementoenamel junction (Fig. 5). The damping ratio showed a trend similar to the PTV. The damping ratio of the upper first premolar and the upper central incisor showed no significant difference (p > 0.05) when the bone level was changed from 2 to 10 mm downward from the cementoenamel junction (Fig. 6). The damping ratios at the 2- and 4-mm bone levels were 15.46 and 22.15%, which were significantly lower than the damping ratios of the upper central incisor and the upper first premolar at the same bone levels (p < 0.05). For the upper central incisor, upper first premolar, and upper first molar, the fold changes in damping ratio from 2 to 10 mm were 1.77, 1.57, and 2.58, respectively.

## Discussion

The objective of this study was to evaluate and compare the correlation between tooth stability parameters, represented by PTV, resonance frequency and damping ratio. In addition, the damping ratio analysis for continuously monitoring the various degrees of vertical alveolar bone loss and different root types were also assessed. The resonance frequency is used for evaluating the stability of natural teeth [19, 21]. Huang et al. [15] found that the mean resonance frequency of anterior teeth significantly decreases in value due to tooth periodontal attachment loss. The results of our study showed that the resonance frequencies of the simulated teeth decreased with the simulated alveolar bone loss (Fig. 2a). This finding is consistent with previous in vivo data [15, 16, 21], which also showed that teeth with periodontal disease exhibited lower resonance frequencies compared to analog teeth with healthy attachment bone levels.

According to previous reports, the elastic modulus of enamel top surface measured between 101 and 117 GPa [39, 40]. Most metals that can be manufactured for 3D printing, such as Co-Cr alloy and stainless steel, have greater elastic moduli than enamel. With this regard, Ti6Al4V (115 GPa) was selected for fabricating the simulated teeth in this study. Since the real damping ratio of the teeth cannot be determined and the results presented above can only reflect the trend of changes in DF values.

In Fig. 4, the resonance frequencies of upper first molars samples are higher than that of upper first premolar and upper central incisor samples. The reason is that the first molar is a three-root tooth, which demonstrates greater stability than the other tooth models with one or two roots. A previous finite element analysis also indicated that RF curves of single-root teeth and multiple-root teeth showed decreasing trends with alveolar bone height loss, and the multiple-root teeth had higher resonance frequencies than single-root teeth did at the same alveolar bone height [22, 38, 41].

The periotest (Siemens) is reported to be an accurate detector for measuring tooth mobility and is used in the diagnostic evaluation of periodontal disease. The PTV is a measure of the displacement amplitude of a tooth upon subjection to an impact loading. For years, the PTV was used to represent the periodontal ligament of the target tooth [14, 42]. Feng et al. utilized resonance frequency and damping ratio in dental implant study and found that when the Young’s modulus of the surrounding bone increased, the implant resonance frequency increased and the damping ratio and PTV values decreased [27–29]. This negative correction phenomenon between the resonance frequency and damping property was also observed in our study (Fig. 3). Molar furcation involvement is an important factor in both biomechanical and clinical periodontology [38]. As shown in Fig. 5, the upper first molar model exhibited lower PTV and damping ratio than other models did. Notably, when the amount of vertical alveolar bone loss increased from 4 to 6 mm apically from the cementoenamel junction, the PTV of the upper first molar model increased remarkably. This increase indicates that the multiple-root teeth had higher stability than single-root teeth, which confirms the findings reported by Doshi et al. [43].

When we evaluated the association of PTVs with the amount of bone and clinical attachment loss, the anterior teeth showed higher PTVs compared with the posterior teeth; and the mandibular teeth showed higher PTVs compared with the maxillary teeth. Interestingly, we also found that when alveolar bone loss exposed a furcation, the tooth stability decreased significantly (Fig. 5). We observed increasingly greater loss of stability with the number of furcations exposed. Generally, trifurcated upper first molars had the best stability, followed by bifurcated upper first premolars, and then furcation less upper central incisors.

Damping property is a phenomenon to describe how oscillations in a system decay after a disturbance. The load response of the viscoelastic tissues can be strain-rate dependent. Since the more is the hysteresis in the stress–strain curve, the greater is the energy dissipation. Thus viscoelastic tissues exhibit higher damping ability [44, 45]. The depth of the embedment may significantly affect the system damping response [46]. The damping ratio is a dimensionless value used to describe damping property of a system. It is used to evaluate the viscoelastic behavior of substances [36, 47]. Damping ratio analysis provides additional information on osseointegration of dental implants [27–29] during the healing period because the trabecular bone is important for developing secondary implant stability due to its higher capacity for new bone formation and remodeling. Additionally, the trabecular bone provides a viscoelastic property at the implant/bone interface. The periodontal ligament is well known for its viscoelastic properties. It is the major damping material of teeth and acts as a shock absorber to minimize external loading [48, 49]. Nevertheless, there is no systemic research about the damping behavior of natural teeth, particularly for progressive vertical alveolar bone height loss. In our studies, as the depth of root embedded in the simulated bone decreased, the amount of bone loss increased, and the damping ratio was increased (Fig. 6). These results conformed to previous research by Todorovska [45] who reported that a system’s damping ratio is larger when the depth of the embedment is smaller.

Based on our results, damping ratio analysis is a sensitive parameter for evaluating various simulated degrees of vertical alveolar bone loss. In the rigid interface, such as tooth ankyloses (tooth to bone contact without a periodontal ligament) resonance frequency analysis shows a clearly identified boundary condition. Nonetheless, when softer tissue is present between the interfaces, like with tooth to bone contact with a periodontal ligament or implant to trabecular bone contact, the damping ratio is a more sensitive parameter for evaluating the softer tissue damping conditions. We believe that it is reasonable to suggest that damping ratio analysis is a sensitive tool in a viscoelastic surrounding, and damping ratio is a reliable measure for monitoring the status of periodontal conditions of different root types as the boundary conditions change.

## Conclusion

These results suggested that DR is a sensitive measure of periodontal status of different root types as boundary conditions are changed. Thus, it is reasonable to suggest that DR analysis is an efficient tool to evaluate the periodontal status of teeth. DR analysis combined with RF analysis results in a more sensitive assessment of changes in the periodontal status than RF or PTV analysis alone.

## Abbreviations

PTV: periotest value
RF: resonance frequency
ANOVA: analysis of variance
DR: damping ratio
UCI: upper central incisor
UFP: upper first premolar
UFM: upper first molar
